# Pickering Emulsions Stabilized by Tea Water-Insoluble Protein Nanoparticles From Tea Residues: Responsiveness to Ionic Strength

**DOI:** 10.3389/fnut.2022.892845

**Published:** 2022-04-26

**Authors:** Zhongyang Ren, Zhongzheng Chen, Yuanyuan Zhang, Xiaorong Lin, Wuyin Weng, Bin Li

**Affiliations:** ^1^College of Ocean Food and Biological Engineering, Jimei University, Xiamen, China; ^2^College of Food Science, South China Agricultural University, Guangzhou, China; ^3^Collaborative Innovation Center of Provincial and Ministerial Co-construction for Marine Food Deep Processing, Dalian, China

**Keywords:** tea residues, plant proteins, Pickering emulsions, ionic strength, rheological properties

## Abstract

Tea water-insoluble protein nanoparticles (TWIPNs) can be applied to stabilize Pickering emulsions. However, the effect of ionic strength (0–400 mmol/L) on the characteristics of Pickering emulsions stabilized by TWIPNs (TWIPNPEs) including volume-averaged particle size (d_4,3_), zeta potential, microstructure and rheological properties is still unclear. Therefore, this work researched the effect of ionic strength on the characteristics of TWIPNPEs. The d_4,3_ of TWIPNPEs in the aquatic phase increased with the increase in ionic strength (0–400 mmol/L), which was higher than that in the SDS phase. Furthermore, the flocculation index of TWIPNPEs significantly (*P* < 0.05) increased from 24.48 to 152.92% with the increase in ionic strength. This could be verified from the microstructure observation. These results indicated that ionic strength could promote the flocculation of TWIPNPEs. Besides, the absolute values of zeta potential under different ionic strengths were above 40 mV in favor of the stabilization of TWIPNPEs. The viscosity of TWIPNPEs as a pseudoplastic fluid became thin when shear rate increased from 0.1 to 100 s^−1^. The viscoelasticity of TWIPNPEs increased with increasing ionic strength to make TWIPNPEs form a gel-like Pickering emulsion. the possible mechanism of flocculation stability of TWIPNPEs under different ionic strengths was propose. TWIPNs adsorbed to the oil-water interface would prompt flocculation between different emulsion droplets under the high ionic strength to form gel-like behavior verified by CLSM. These results on the characteristics of TWIPNPEs in a wide ionic strength range would provide the theoretical basis for applying Pickering emulsions stabilized by plant proteins in the food industry.

## Introduction

Emulsions are easily affected by external factors such as pH, temperature and ionic strength to lose stability (1, 2). In recent 10 years, numerous food-grade particles have been developed with the rise of food-grade Pickering emulsions. The development and application of plant proteins have aroused the interest of researchers to structure Pickering emulsions (3). Plant protein particles can combine oil and water to form stable emulsions (4, 5). However, some protein particles only stabilize Pickering emulsions under a certain ionic strength (6).

Protein particles like nanoparticles, microgels and fibrils can manipulate the properties of Pickering emulsions like aggregation or flocculation with the change of ionic strength (7). Numerous raw proteins can form nanoparticles using simple methods. The interfacial behavior of protein nanoparticles from zeins, kafirins, soy proteins, wheat proteins, wheat protein/xanthan gum complexes in Pickering emulsions can be adjusted by ionic strength (8–11). Zein nanoparticles can produce stable o/w Pickering emulsions under moderate ionic strengths (1–10 mmol/L) (8). Protein microgels with soft and deformable properties can quickly swell and deswell reversibly under various external stimuli like ionic strength to form stimuli-responsive emulsions (12). Lots of protein nanoparticles need to be processed by thermal denaturation method, ion bridging method, solvent/antisolvent method to prepare stable Pickering emulsions. These protein nanoparticles must be carefully designed to reach the appropriate size and interface behavior before effectively stabilizing Pickering emulsions (3). Additionally, the performance of protein nanoparticles can be also improved by adjusting ionic strength in the protein nanoparticle solution to suitable interfacial properties (13). Pickering emulsions have been prepared by combining different protein nanoparticles with polysaccharides like gluten nanoparticle/xanthan gum complexes under suitable ionic strengths (11).

Tea proteins from tea residues account for 20–30% of dry tea are mainly tea water-insoluble proteins after the treatment of alkaline method (14) or enzyme method (15). Tea water-insoluble protein nanoparticles (TWIPNs) from tea residues include the uncharged amino acids and hydrophobic amino acids of more than 60% of amino acids gained by the alkali-solution and acid-precipitation method (16). At an oil-water ratio of 6:4, TWIPNs can be used to stabilize Pickering emulsions on the neutral condition, (17). Besides, TWIPNs can be applied to generate gel-like Pickering emulsions after high-pressure homogenization (18). Meanwhile, the flocculation of Pickering emulsions stabilized by TWIPNs (TWIPNPEs) is accelerated at high temperatures (100°C/120°C) (19). Furthermore, TWIPNPEs can exhibit a gel-like behavior at pH 7–11 (20) and as a template to prepare oil gels (21). However, few studies have systematically characterized the properties and colloidal behavior of TWIPNPEs under different ionic strengths. it is very necessary to characterize the ability of TWIPNPEs for responding to the environmental condition of ionic strength.

Therefore, this study aimed to reveal the effect of ionic strength (0–400 mmol/L) on the characteristics of TWIPNPEs under neutral conditions. The characteristics of TWIPNPEs under different ionic strengths were analyzed. We hypothesized that TWIPNs at the interface of oil and water would prompt the flocculation between different emulsion droplets under a high ionic strength, forming gel-like behavior. The gel-like behavior of TWIPNPEs was explored under different ionic strengths.

## Materials and Methods

### Materials

Tea residues were gained from Shenzhen Shenbao Huacheng Tech. Co., Ltd. Soy oil was purchased from the local supermarket in Guangzhou of China. Sodium dodecyl sulfate (SDS, ≥99.0%) was purchased from Merck & Co., Inc. Other reagents were of analytical purity. All water was of deionized water.

### Preparation of TWIPNs

TWIPNs were prepared according to a previous method (17). Briefly, tea residues (100 g) were extracted at 90°C for 1.5 h using a 3 L NaOH solution (0.3 mol/L) and centrifuged at 8,288 g for 15 min at 25°C. The supernatant was precipitated at pH 3.5 after decolorization using 30% H_2_O_2_. TWIPNs were gained after washing the precipitates to the neutral and frozen-drying via a freeze drier (Christ, ALPHA 1-2 LD Plus, Osterode, Germany). The TWIPNs (2 g) were dispersed with the different NaCl solutions (0–400 mmol/L) and hydrated for 24 h at 4°C for preparing emulsions.

### Preparation of TWIPNPEs

TWIPNPEs were prepared according to a previous method (21). TWIPN suspension (40 mL) and soy oils (60 mL) were mixed using a shear emulsifying machine (SUOTN, AD500S-H, Shanghai, China) at 20,000 r/min for 2 min to form initial emulsions. The initial emulsions were homogenized at 40 MPa by a high-pressure homogenizer (AH-Basic-II, Suzhou, China) to obtain TWIPNPEs.

### Measurements of Droplet Size and Flocculation Index of TWIPNPEs

The volume-average droplet size (d_4,3_) and flocculation index of TWIPNPEs were determined by Mastersizer (Malvern 3000, Malvern, UK) according to a previous method (22). Water and 10 g/L SDS solution were used as dispersants. The flocculation index was calculated according to Eq. (1).


(1) $$\begin{array}{l} \text{FI}\left(\text{\%}\right)=\left({\text{d}}_{4,3-\text{W}}/{\text{d}}_{4,3-\text{S}}-1\right)\times 100 \end{array}$$

where d_4,3−W_ and d_4,3−S_ represent the droplet size of emulsions in water and SDS dispersion, respectively.

### Measurement of Zeta Potential of TWIPNPEs

The zeta potential of TWIPNPE was measured according to a previous method (23). TWIPNPEs (10 μL) were mixed using water (990 μL). The Malvern zetasizer (Nano ZS90, Malvern, UK) was equilibrated for 60 s after putting a disposable folded capillary cell. The testing condition was performed at 25°C.

### Morphological Observation of TWIPNPEs

TWIPNPEs were observed via an optical microscope (Motic, BA310-T, Hong Kong, China) equipped with a 100 × lens. Then, the confocal laser scanning microscope (CLSM) with LSM 7 DUO dual confocal system was used to observe TWIPNPEs. The emulsions were stained before observation with Nile blue A for TWIPNs and Nile red for soybean oil. The TWIPNPEs were stained with anhydrous ethanol containing 0.05% Nile blue A and 0.05% Nile red for 30 min. TWIPNPE samples were observed by Argon and He/Ne lasers with the excitation wavelengths of 488 nm and 633 nm.

### Determination of Rheological Properties of TWIPNPEs

TWIPNPEs were determined by rheometer (Anton Paar, MCR-102, Graz, Austria) according to a previous method (24). The apparent viscosity of TWIPNPEs was analyzed at shear rates from 0.1 to 100 s^−1^ using a parallel plate (Φ50 mm). The frequency sweep of TWIPNPEs was tested at the angular velocity from 0.1 to 100 rad/s within a small amplitude oscillatory mode. The gap was fixed at 1.0 mm. All the tests were performed at 25 ± 0.1°C. The storage modulus (G') and loss modulus (G") were recorded.

### Statistical Analysis

Data were expressed as the mean values ± standard deviation. Origin Pro 9.0.5 software was used to analyze the significance (*P* < 0.05).

## Results and Discussion

### Stability of Pickering Emulsions Prepared Using TWIPNs Under Different Ionic Strengths

#### Droplet Size

**Figure 1:**
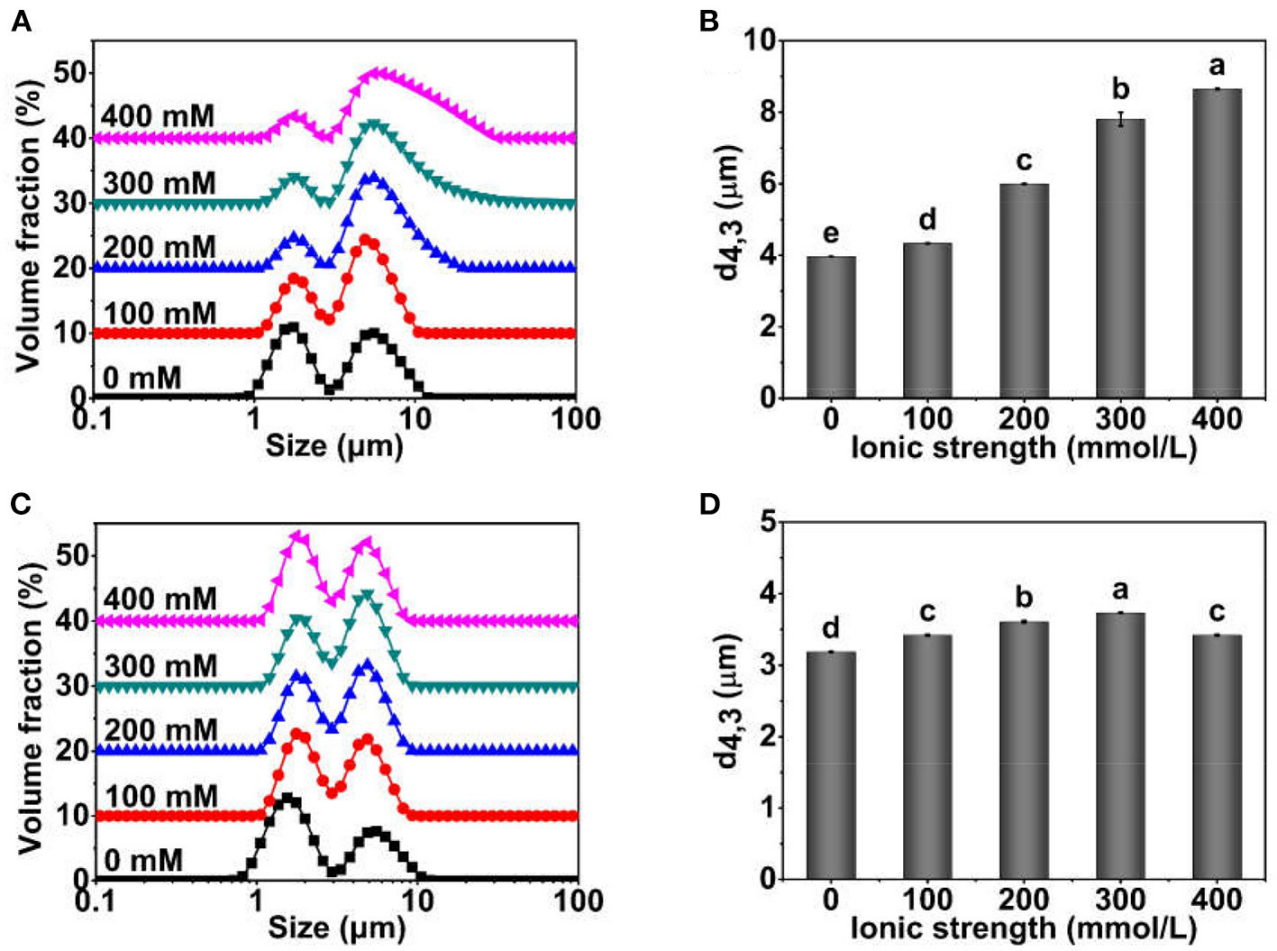
Particle size distribution profiles and d_4,3_ of Pickering emulsions stabilized by TWIPNs at different ionic strengths [**(A)** Particle size distribution in water; **(B)** d_4,3_ in water; **(C)** Particle size distribution in 1.0% SDS; **(D)** d_4,3_ in 1.0% SDS]. The lowercases indicate the significance of different Pickering emulsions by tea water-insoluble protein nanoparticles under different ionic strengths (*P* < 0.05).

**Figure 2:**
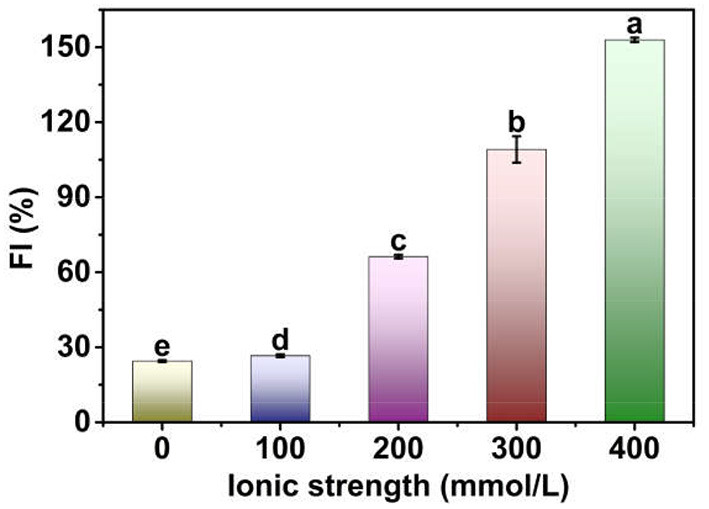
FI of Pickering emulsions stabilized by tea water-insoluble protein nanoparticles under different ionic strengths. The lowercases indicate the significance of different Pickering emulsions by tea water-insoluble protein nanoparticles under different ionic strengths (*P* < 0.05).

The particle size distribution and d_4,3_ of TWIPNPEs using TWIPNs under different ionic strengths are shown in Figure 1. The particle size distribution of TWIPNPEs in the aqueous phase presented two peaks. The volume fraction of peak 1 decreased with the increase in ionic strength, while the volume fraction of peak 2 increased, indicating that the droplets of TWIPNPEs in the aquatic phase increased with the increase in ionic strength (Figure 1A). The d_4,3_ of TWIPNPEs in the aquatic phase increased with the increase from 0 to 400 mmol/L NaCl (Figure 1B), which was consistent with the particle size distribution in Figure 1A. It also has been reported that the size of emulsions stabilized by pea protein isolate nanoparticles increases with the increase in the ionic strength (25). The increase in droplet size of emulsions with the increase in ionic strength might be due to that the strong electrostatic screening induces coalescence (26). In addition, the particle size distribution and d_4,3_ of TWIPNPEs dispersing in 1.0% SDS indicated that the particle size of TWIPNPEs increased with the increase of ionic strengths (0–300 mmol/L) and decreased under the ionic strength of 400 mmol/L. The d_4,3_ of TWIPNPEs in 1.0% SDS was significantly smaller than those in the aquatic phase. This may result in the flocculation of Pickering emulsions, which could be verified by FI in Figure 2.

#### Flocculation Index

The FI of TWIPNPEs under different ionic strengths is shown in Figure 2. The FI of TWIPNPEs significantly (*P* < 0.05) increased from 24.48 to 152.92% with the increase in ionic strength. These results indicated that ionic strength could promote the flocculation of TWIPNPEs under the ionic strengths of 0–400 mmol/L. It has been indicated that the droplet flocculation of Pickering emulsions stabilized by pea protein microgel particles can be observed after the addition of 100 mM NaCl (27). This can be attributed to that protein particles interact with each other at the surface of emulsion droplets with the increase in ionic strength, thereby increasing the flocculation of Pickering emulsions (28). Besides, salt ions can be applied to control the electrostatic interactions between the droplets of Pickering emulsions and affect the formation of emulsion gels (29). Meanwhile, ionic strength promoted the formation of aggregates of TWIPNs to increase the size of protein particles, which increased the aggregation of adjacent TWIPNs to improve the flocculation of emulsion droplets.

#### Microscope Observation

**Figure 3:**
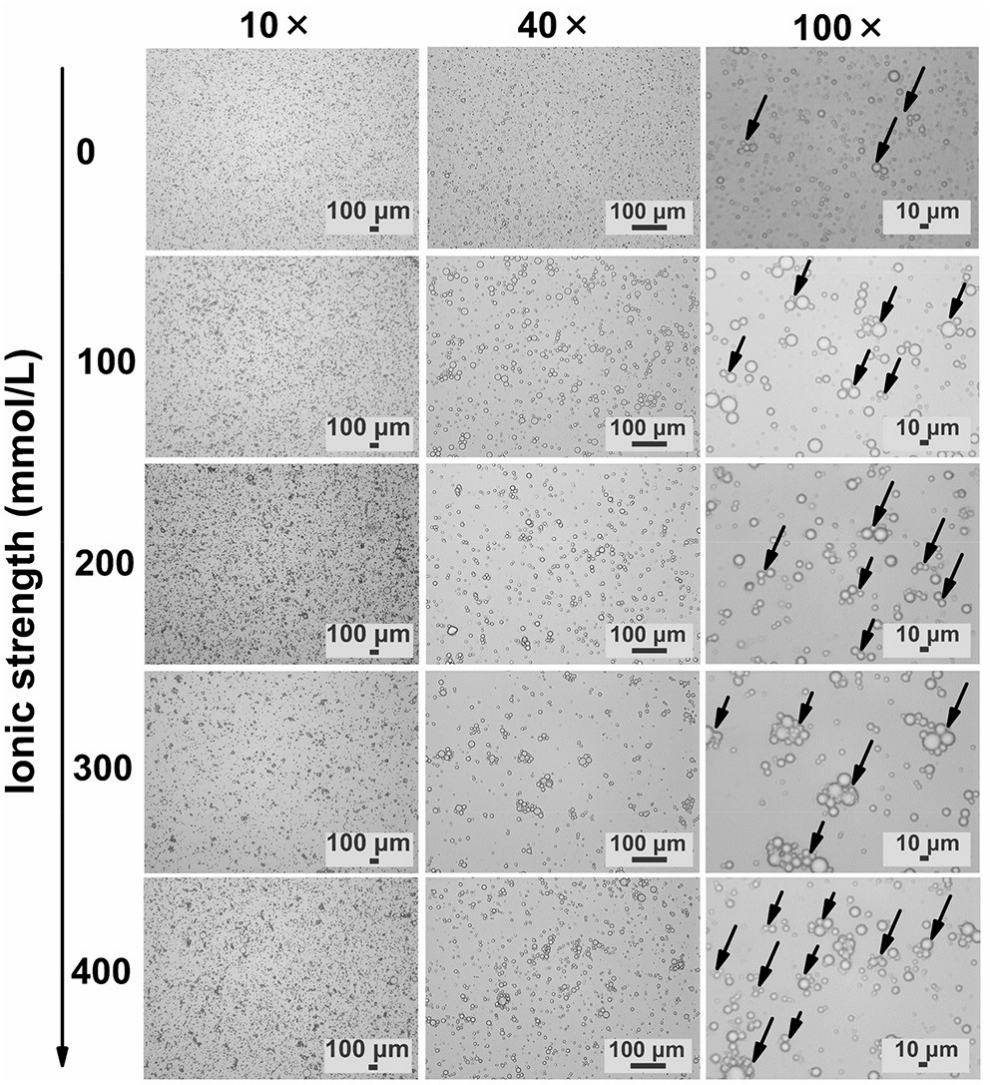
Microscopic observation of Pickering emulsions stabilized by tea water-insoluble protein nanoparticles under different ionic strengths (left: ionic strength; upper: magnification times. Arrows indicate the flocculation).

TWIPNPEs under different ionic strengths were further observed as shown in Figure 3. As indicated by the black arrow, the flocculation of emulsion droplets gradually increased with increasing ionic strength. This result was in agreement with the analysis of particle size (Figure 1) and flocculation of TWIPNPEs under different ionic strengths (Figure 2). According to the observation of TWIPNPEs in 100 × objective lens in Figure 3, it could be observed that the flocculation of TWIPNPEs increased from 0 to 400 mmol/L NaCl as indicated using the arrows. This indicates the network formation of aggregated emulsion droplets among the adjacent emulsion droplets. The formation of inter-droplet particle networks at the high ionic strengths is verified like Pickering emulsions stabilized by zein (8), hydrophilically modified silica nanoparticles (30), silica nanoparticles coated with (3-glycidyloxypropyl) trimethoxysilane (31) and so on. Protein nanoparticles can be induced to form a network through salt ions.

#### Zeta Potential

**Figure 4:**
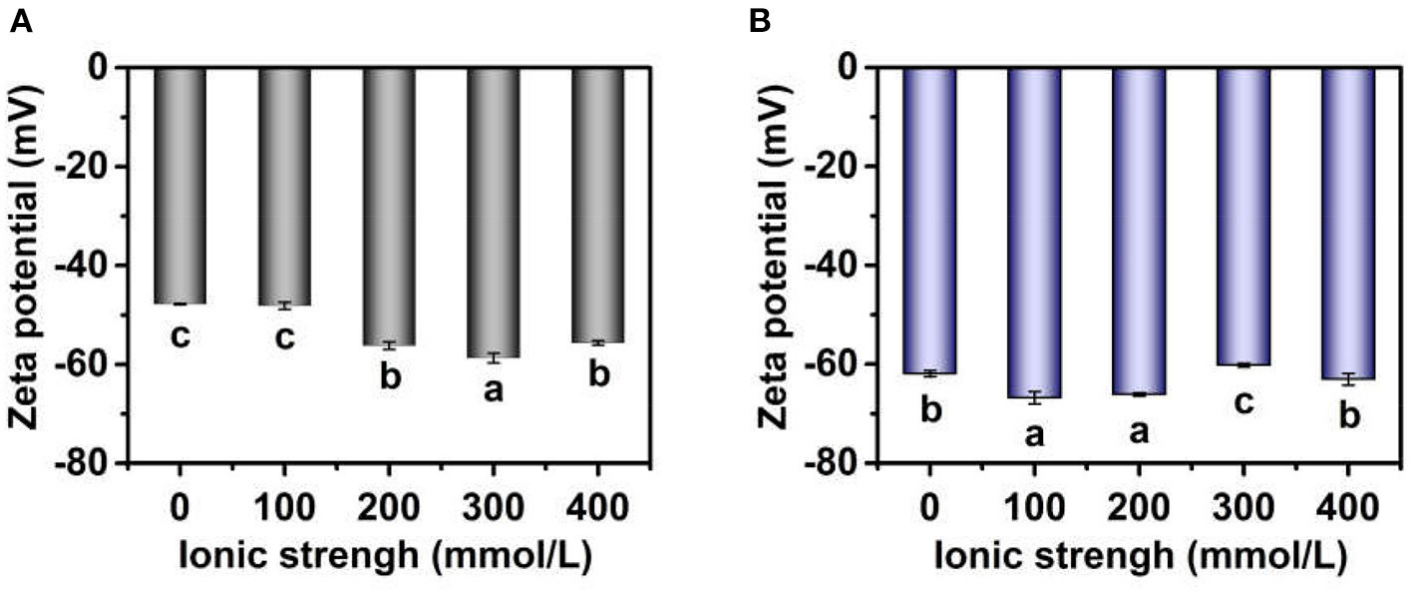
Zeta potential of Pickering emulsions by tea water-insoluble protein nanoparticles under different ionic strengths [**(A)** emulsions in water; **(B)** emulsions in 1.0% SDS]. The lowercases indicate the significance of different Pickering emulsions by tea water-insoluble protein nanoparticles under different ionic strengths (*P* < 0.05).

The zeta potential of TWIPNPEs under different ionic strengths was also analyzed as shown in Figure 4. The zeta potential of TWIPNPEs under the ionic strength of 100 mmol/L had no significant change (*P* > 0.05) compared with that without the addition of salt (Figure 4A). However, the absolute zeta potential value of TWIPNPEs without SDS treatment increased with increasing ionic strength at above 100 mmol/L, which was higher than that under the ionic strength of no more than 100 mmol/L. In this study, the emulsions were prepared at pH 7, which is above the isoelectric point (pH 3.5) of TWIPNs (16). the zeta potential of the emulsions stabilized by proteins at pH above the isoelectric point with negative charges increases with the increase in ionic strength (32). When the ionic strength reached 400 mmol/L, the zeta potential of TWIPNPEs showed a slight decrease compared to that under the ionic strength of 300 mmol/L (Figure 4A). This may be attributed to the flocculation of TWIPNPE droplets under the high ionic strength, leading to the reduction of exposing charged residue groups at the surface of emulsion droplets. The decrease in negative charges of emulsions stabilized by proteins at pH above the isoelectric point could be attributed to electrostatic screening and ion binding effects (32, 33). The absolute value of zeta potential of TWIPNPEs in the SDS solution was higher than those in the deionized water as shown in Figure 4B. It is due to that SDS can destroy the emulsion flocculation, resulting in more exposure of anions at the droplet surface. The absolute values of zeta potential of TWIPNPEs under different ionic strengths were above 40 mV, which was beneficial to the stabilization of TWIPNPEs according to previous reports (17, 34).

### Rheological Behavior of Pickering Emulsions Prepared Using TWIPNs Under Different Ionic Strengths

**Figure 5:**
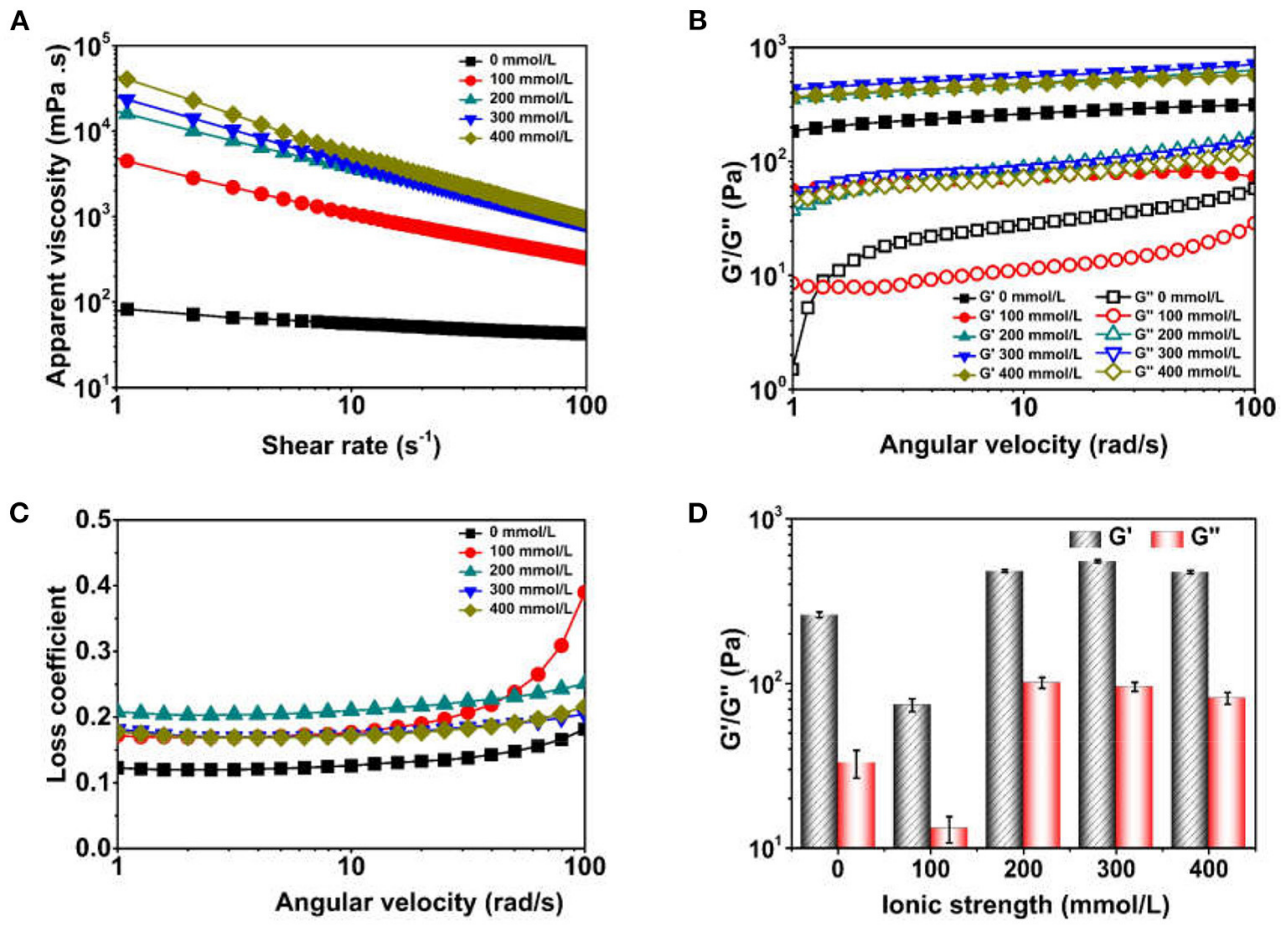
Apparent viscosity and viscoelastic parameter of Pickering emulsions by tea water-insoluble protein nanoparticles under different ionic strengths [**(A)** apparent viscosity; **(B)** G'/G”; **(C)** Loss coefficient; **(D)** G'/G” at 10 rad/s].

Ionic strength can also affect the rheological behavior of Pickering emulsions except for the particle size, zeta potential, flocculation of emulsions. The rheological behavior of TWIPNPEs under different ionic strengths is presented in Figure 5. The apparent viscosity of TWIPNPEs under different ionic strengths decreased with the increase in shear rate (Figure 5A), which showed the TWIPNPEs belonged to the pseudoplastic fluid as a previous report (35). The TWIPNPEs at the same shear rate became thick with the increase in ionic strength. Meanwhile, the apparent viscosity of TWIPNPEs increased under the ionic strength from 0 to 400 mmol/L at the same angular velocity. The particle size, flocculation and droplet interaction increased with the increase in ionic strength, resulting in the improvement of the apparent viscosity of TWIPNPEs (Figures 1, 2). Besides, the thick emulsions under high ionic strengths contain no free solid particles in the emulsion system due to the aggregation of emulsion droplets together with each other (1).

The G', G”and loss coefficient of TWIPNPEs stabilized by TWIPNs at different ionic strengths were analyzed (Figures 5B,C). In the linear viscoelastic range, the G' and G” values of TWIPNPEs decreased below the ionic strength of 100 mmol/L at the same angular velocities. However, the G' and G” values of TWIPNPEs under the ionic strength of more than 200 mmol/L were similar, which was higher than that of TWIPNPEs under the ionic strength below 100 mmol/L. The G' values of TWIPNPEs were higher than G”, indicating that the TWIPNPEs under different ionic strengths possessed the gel-like behavior as previous reports (36). The loss coefficient of TWIPNPEs was no more than 0.5 (Figure 5C), implying that these emulsions possessed dominant elasticity to form gel-like behavior. The G' values of TWIPNPEs were much higher than G”. As previously reported, this is beneficial for the formation of gel-like networks (37, 38). The viscoelasticity of TWIPNPEs could be also obtained at 10 rad/s (Figure 5D). However, Destroying the gel-like behavior of emulsions using shearing at high yield stress may induce the emulsion coalescence due to the network of flocculated solid particles (39). As shown in (Figure 5A), the viscosity of TWIPNPEs as a pseudoplastic fluid became thin when the shear rate increased from 0.1 to 100 s^−1^. The yield stress of pseudoplastic fluid is increased with an increase in shear rate (40). According to previously reported, a suitable ionic strength can control the yield stress to reach the workability of emulsions with respect to its practical applications in the food industry (1).

### Possible Mechanism of Pickering Emulsions Prepared Using TWIPNs Under Different Ionic Strengths

The addition of salt ions can change the ionic strength of the solution. Monovalent or polyvalent metal ions could effectively reduce the electrostatic shielding by reducing the dipole moment, thus reducing the zeta potential on the surface of the emulsion droplets to affect the emulsion stability (8). Monovalent, divalent and trivalent metal ions prompt the flocculation of different kinds of emulsions; meanwhile, univalent metal ion salts such as NaCl are usually used to analyze the effect of ionic strength on emulsion properties (41, 42). The change of ionic strength results in the different flocculation degrees of emulsions. In this research, ionic strengths of 0–400 mmol/L were chosen to cause a certain amount of flocculation (Figure 2), which could improve the stability to some extent rather than destabilization of TWIPNPEs. As a previous report, walnut protein/xanthan gum complexes under the ionic strength of 500 mmol/L NaCl increase the degree of dissipative flocculation (43). A certain quantity of salt ions in the water phase inhibits electrostatic repulsion between protein nanoparticles to promote closer packing at the interface of emulsion droplets (44). The presence of a certain amount of salt ions is crucial for forming stable Pickering emulsions (45).

**Figure 6:**
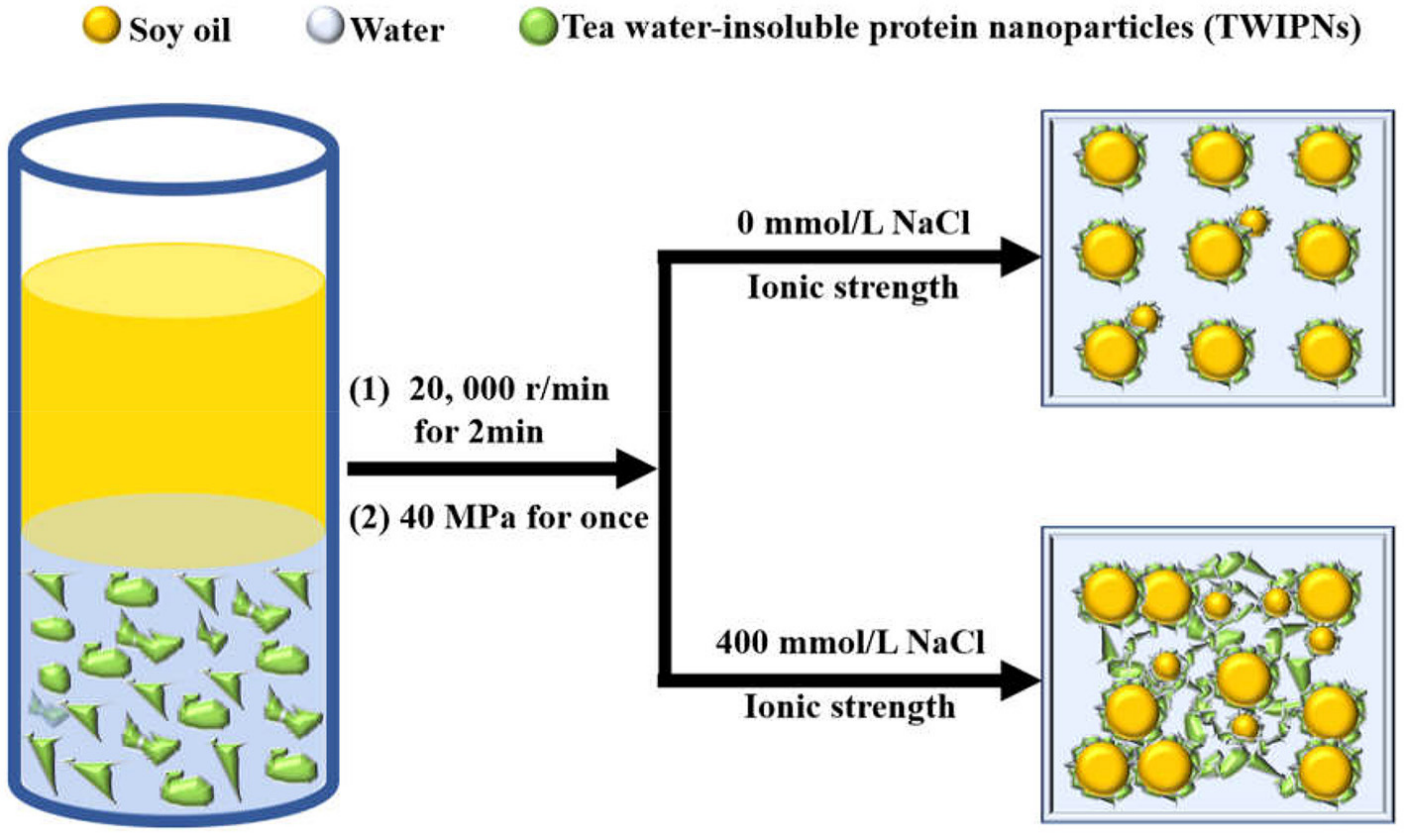
Schematic diagram of flocculation stability of Pickering emulsions by tea water-insoluble protein nanoparticles under different ionic strengths.

**Figure 7:**
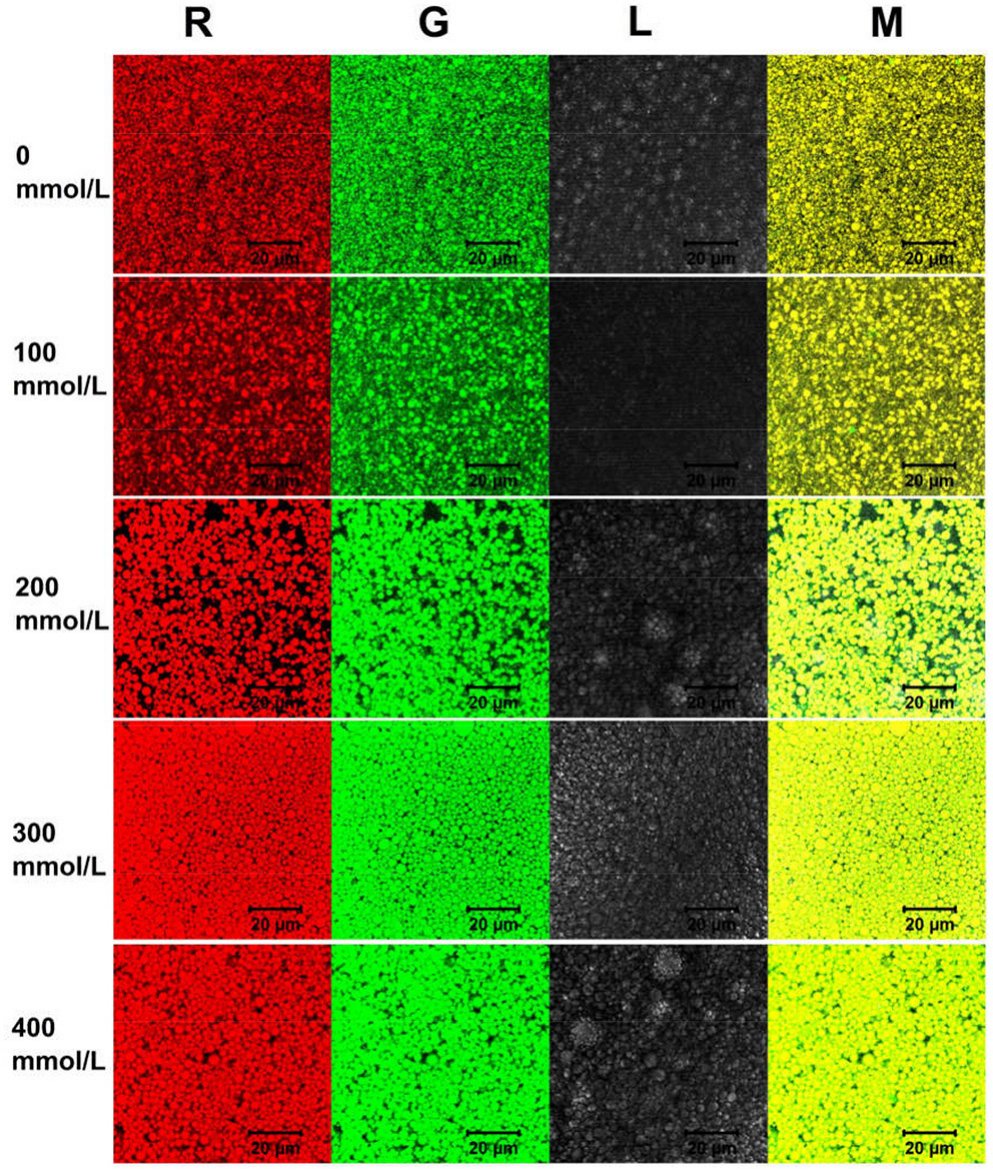
Confocal laser scanning microscopy images of Pickering emulsions by tea water-insoluble protein nanoparticles under different ionic strengths.

Here, the schematic diagram of flocculation stability of TWIP stabilized Pickering emulsions under different ionic strengths is shown in Figure 6. The viscosity and viscoelasticity of TWIPNPEs increased under the ionic strength from 0 to 400 mmol/L to benefit for forming gel-like TWIPNPEs (Figures 5A,B). The selection of appropriate ionic strength can control the yield stress, contributing to that Pickering emulsions meet the practical application of processability in the food industry (1). TWIPNs flocculated with each other to form gel-like stable emulsions in a certain concentration of ionic strengths (Figure 5B). The microstructures of TWIPNPEs under different ionic strengths of 0–400 mmol/L were also investigated by CLSM (Figure 7). A reinforcement of the flocculation of the TWIPNPEs was verified with the increase of ionic strengths, especially above 200 mmol/L. This is consistent with a previous report about the flocculated network due to the increase in ionic strengths (46). The formation of a gel-like network in TWIPNPEs was certainly due to relate with ionic strengths in this study. However, it should be carefully considered that shearing could damage the gel system above the yield stress to destruct gel-like structures of Pickering emulsions, leading to the coalescence and further instability of Pickering emulsions (39).

When salt is added to the nanoparticle dispersion, salt ions are selectively adsorbed at the particle surface to neutralize the net charge to reduce the number of charges around the nanoparticles and the thickness of the double electric layer, furtherly forming the flocculation of emulsions (1). The valence of metal ions is closely related to the flocculation of emulsions. The flocculation degree of divalent and trivalent metal ions is more than approximately 10–100 times that of univalent metal ions at the same ionic strength (47). Within a certain range, improving ionic strength can increase the hydrophobicity of particles to promote the adsorption of particles at the oil and water interface (48). An appropriate amount of ionic strength can also reduce the electrostatic repulsion between particles, which is conducive to the accumulation of particles at the surface of emulsion droplets to form a layer (44). Protein particles effectively adsorbed to the surface of the emulsion droplets result in the change of the dependence of emulsion droplets on ionic strength, thus allowing emulsions to achieve the flocculation stability in a suitable ionic strength range (17).

## Conclusion

This work researched the effect of ionic strength on the characteristics of TWIPNPEs under neutral conditions. First of all, the d_4,3_ of TWIPNPEs in the aquatic phase increased more than that in the SDS phase. Besides, ionic strength promoted the aggregation of TWIPNs to increase the size of protein particles for improving the flocculation of emulsion droplets through the microstructure observation. With the increase in ionic strength, the apparent viscosity and viscoelasticity of TWIPNPEs increased, especially when the ionic strength was above 200 mmol/L, showing good gel-like properties. This was consistent with our hypothesis that TWIPNs adsorbed to the oil-water interface would prompt flocculation between different emulsion droplets under the high ionic strength to form gel-like behavior. These findings can be utilized in the food industry for improving the stabilization of Pickering emulsions stabilized by tea proteins from tea residues under a wide range of ionic strengths to broaden the utility and application of Pickering emulsions in different environments of food manufacture.

## Data Availability Statement

The raw data supporting the conclusions of this article will be made available by the authors, without undue reservation.

## Author Contributions

ZR: data curation, formal analysis, investigation, methodology, resources, software, validation, visualization, and writing—original draft. ZC: data curation, formal analysis, and writing—original draft. YZ: data curation, formal analysis, and methodology. XL: validation, visualization, and writing—original draft. WW: formal analysis and writing—review and editing. BL: funding acquisition, project administration, supervision, and writing—review and editing. All authors contributed to the article and approved the submitted version.

## Funding

This study was funded by the Natural Science Foundation of Fujian Province in China (2021J01835), National Key Research and Development Program of China (2021YFD210020204), Fujian Science and Technology Project (2021N5013), Special Fund from the Modern Agricultural Industry of China (CARS-19), and the Scientific Research Foundation of Jimei University in China (ZQ2020011).

## Conflict of Interest

The authors declare that the research was conducted in the absence of any commercial or financial relationships that could be construed as a potential conflict of interest.

## Publisher's Note

All claims expressed in this article are solely those of the authors and do not necessarily represent those of their affiliated organizations, or those of the publisher, the editors and the reviewers. Any product that may be evaluated in this article, or claim that may be made by its manufacturer, is not guaranteed or endorsed by the publisher.

## References

[1] Chevalier Y Bolzinger MA. Emulsions stabilized with solid nanoparticles: Pickering emulsions. Colloids Surf A. (2013) 439:23–34. 10.1016/j.colsurfa.2013.02.05431631200

[2] Li S Zhang B Li C Fu X Huang Q. Pickering emulsion gel stabilized by octenylsuccinate quinoa starch granule as lutein carrier: role of the gel network. Food Chem. (2020) 305:125476. 10.1016/j.foodchem.2019.125476 31525589

[3] Xiao J Li Y Huang Q. Recent advances on food-grade particles stabilized Pickering emulsions: fabrication, characterization and research trends. Trends Food Sci Tech. (2016) 55:48–60. 10.1016/j.tifs.2016.05.010

[4] Zhu X Zheng J Liu F Qiu C Lin W Tang C. Freeze-thaw stability of Pickering emulsions stabilized by soy protein nanoparticles. Influence of ionic strength before or after emulsification. Food Hydrocoll. (2018) 74:37–45. 10.1016/j.foodhyd.2017.07.017

[5] Zhou F Yu X Zeng T Yin S Tang C Yang X. Fabrication and characterization of novel water-insoluble protein porous materials derived from Pickering high internal-phase emulsions stabilized by gliadin-chitosan-complex particles. J Agr Food Chem. (2019) 67:3423–31. 10.1021/acs.jafc.9b00221 30835109

[6] McClements DJ. Protein-stabilized emulsions. Curr Opin Colloid In. (2004) 9:305–13. 10.1016/j.cocis.2004.09.003

[7] Bertoncarabin CC Schroën K. Pickering emulsions for food applications: background, trends, and challenges. Annu Rev Food Sci T. (2015) 6:263–97. 10.1146/annurev-food-081114-110822 25705932

[8] De Folter JW van Ruijven MW Velikov KP. Oil-in-water Pickering emulsions stabilized by colloidal particles from the water-insoluble protein zein. Soft Matter. (2012) 8:6807–15. 10.1039/c2sm07417f

[9] Xiao J Wang X Gonzalez AJP Huang Q. Kafirin nanoparticles-stabilized Pickering emulsions: Microstructure and rheological behavior. Food Hydrocoll. (2016) 54:30–9. 10.1016/j.foodhyd.2015.09.008

[10] Zhu YQ Chen X McClements DJ Zou L Liu W. Pickering-stabilized emulsion gels fabricated from wheat protein nanoparticles: Effect of pH, NaCl and oil content. J Disper Sci Technol. (2018) 39:826–35. 10.1080/01932691.2017.1398660

[11] Fu D Deng S McClements DJ Zhou L Zou L Yi J. Encapsulation of β-carotene in wheat gluten nanoparticle-xanthan gum-stabilized Pickering emulsions: enhancement of carotenoid stability and bioaccessibility. Food Hydrocoll. (2019) 89:80–9. 10.1016/j.foodhyd.2018.10.032

[12] Yan X Ma C Cui F McClements DJ Liu X Liu F. Protein-stabilized Pickering emulsions: formation, stability, properties, and applications in foods. Trends Food Sci Tech. (2020) 103:293–303. 10.1016/j.tifs.2020.07.005

[13] Dai L Sun C Wei Y Zhan X Mao L Gao Y. Formation and characterization of zein-propylene glycol alginate-surfactant ternary complexes: effect of surfactant type. Food Chem. (2018) 258:321–30. 10.1016/j.foodchem.2018.03.077 29655740

[14] Zhang C Bozileva E Klis FVD Dong Y Sanders JPM Bruins ME. Integration of galacturonic acid extraction with alkaline protein extraction from green tea leaf residue. Ind Crop Prod. (2016) 89:95–102. 10.1016/j.indcrop.2016.04.074

[15] Shen L Wang X Wang Z Wu Y Chen J. Studies on tea protein extraction using alkaline and enzyme methods. Food Chem. (2008) 107:929–38. 10.1016/j.foodchem.2007.08.047

[16] Ren Z Chen Z Zhang Y Zhao T Ye X Gao X. Functional properties and structural profiles of water-insoluble proteins from three types of tea residues. LWT-Food Sci Technol. (2019) 110:324–31. 10.1016/j.lwt.2019.04.101

[17] Ren Z Chen Z Zhang Y Lin X Li B. Novel food-grade Pickering emulsions stabilized by tea water-insoluble protein nanoparticles from tea residues. Food Hydrocoll. (2019) 96:322–30. 10.1016/j.foodhyd.2019.05.015

[18] Ren Z Chen Z Zhang Y Lin X Li B. Characteristics and rheological behavior of Pickering emulsions stabilized by tea water-insoluble protein nanoparticles via high-pressure homogenization. Int J Biol Macromol. (2020) 151:247–56. 10.1016/j.ijbiomac.2020.02.090 32057881

[19] Ren Z Chen Z Zhang Y Lin X Li Z Weng W. Effect of heat-treated tea water-insoluble protein nanoparticles on the characteristics of Pickering emulsions. LWT-Food Sci Technol. (2021) 149:111999. 10.1016/j.lwt.2021.111999

[20] Ren Z Chen Z Zhang Y Lin X Weng W Liu G. Characteristics of Pickering emulsions stabilized by tea water-insoluble protein nanoparticles at different pH values. Food Chem. (2022) 375:131795. 10.1016/j.foodchem.2021.131795 34922274

[21] Ren Z Li Z Chen Z Zhang Y Lin X Weng W. Characteristics and application of fish oil-in-water Pickering emulsions structured with tea water-insoluble proteins/κ-carrageenan complexes. Food Hydrocoll. (2021) 114:106562. 10.1016/j.foodhyd.2020.106562

[22] Liu F Tang CH. Soy glycinin as food-grade Pickering stabilizers: Part. I structural characteristics, emulsifying properties and adsorption/arrangement at interface. Food Hydrocoll. (2016) 60:606–19. 10.1016/j.foodhyd.2015.04.025

[23] Xue J Wang T Hu Q Zhou M Luo Y. Insight into natural biopolymer-emulsified solid lipid nanoparticles for encapsulation of curcumin: effect of loading methods. Food Hydrocoll. (2018) 79:110–6. 10.1016/j.foodhyd.2017.12.018

[24] Feng X Tjia JYY Zhou Y Liu Q Fu C Yang H. Effects of tocopherol nanoemulsion addition on fish sausage properties and fatty acid oxidation. LWT. (2020) 118:108737. 10.1016/j.lwt.2019.108737

[25] Li X Liu W Xu B Zhang B. Simple method for fabrication of high internal phase emulsions solely using novel pea protein isolate nanoparticles: stability of ionic strength and temperature. Food Chem. (2022) 370:130899. 10.1016/j.foodchem.2021.130899 34509149

[26] Zhong Y Xiang X Wang X Zhang Y Hu M Chen T. Fabrication and characterization of oil-in-water emulsions stabilized by macadamia protein isolate/chitosan hydrochloride composite polymers. Food Hydrocoll. (2020) 103:105655. 10.1016/j.foodhyd.2020.105655

[27] Zhang S Holmes M Ettelaie R Sarkar A. Pea protein microgel particles as Pickering stabilisers of oil-in-water emulsions: Responsiveness to pH and ionic strength. Food Hydrocoll. (2020) 102:105583. 10.1016/j.foodhyd.2019.105583

[28] Dickhout JM Kleijn JM Lammertink R De WV. Adhesion of emulsified oil droplets to hydrophilic and hydrophobic surfaces-effect of surfactant charge, surfactant concentration and ionic strength. Soft Matter. (2018) 14:5452–60. 10.1039/C8SM00476E 29911238

[29] Zhu Y McClements DJ Zhou W Peng S Zhou L Zou L. Influence of ionic strength and thermal pretreatment on the freeze-thaw stability of Pickering emulsion gels. Food Chem. (2020) 303:125401. 10.1016/j.foodchem.2019.125401 31466031

[30] Griffith C Daigle H. Manipulation of Pickering emulsion rheology using hydrophilically modified silica nanoparticles in brine. J Colloid Interf Sci. (2018) 509:132–9. 10.1016/j.jcis.2017.08.100 28898733

[31] Hatchell D Song W Daigle H. Examining the role of salinity on the dynamic stability of Pickering emulsions. J Colloid Interf Sci. (2022) 608:2321–9. 10.1016/j.jcis.2021.10.154 34809989

[32] Sriprablom J Luangpituksa P Wongkongkatep J Pongtharangkul T Suphantharika M. Influence of pH and ionic strength on the physical and rheological properties and stability of whey protein stabilized o/w emulsions containing xanthan gum. J Food Eng. (2019) 242:141–52. 10.1016/j.jfoodeng.2018.08.031

[33] Griffin K Khouryieh H. Influence of electrostatic interactions on the formation and stability of multilayer fish oil-in-water emulsions stabilized by whey protein-xanthan-locust bean complexes. J Food Eng. (2020) 277:109893. 10.1016/j.jfoodeng.2019.109893

[34] Dickinson E. Hydrocolloids as emulsifiers and emulsion stabilizers. Food Hydrocoll. (2009) 23:1473–82. 10.1016/j.foodhyd.2008.08.005

[35] Kumar Y Roy S Devra A Dhiman A Prabhakar PK. Ultrasonication of mayonnaise formulated with xanthan and guar gums: Rheological modeling, effects on optical properties and emulsion stability. LWT-Food Sci Technol. (2021) 149:111632. 10.1016/j.lwt.2021.111632

[36] Yang D Gao S Yang H. Effects of sucrose addition on the rheology and structure of iota-carrageenan. Food Hydrocoll. (2020) 99:105317. 10.1016/j.foodhyd.2019.10531733205393

[37] Yang Z Yang H Yang H. Effects of sucrose addition on the rheology and microstructure of κ-carrageenan gel. Food Hydrocoll. (2018) 75:164–73. 10.1016/j.foodhyd.2017.08.03233205393

[38] Wang JJ Wang Y Wang Q Yang J Hu S Chen L. Mechanically strong and highly tough prolamin protein hydrogels designed from double-cross-linked assembled networks. Acs Appl Mater Inter. (2019) 1:1272–9. 10.1021/acsapm.9b00066

[39] Whitby CP Fischer FE Fornasiero D Ralston J. Shear-induced coalescence of oil-in-water Pickering emulsions. J Colloid Interf Sci. (2011) 361:170–7. 10.1016/j.jcis.2011.05.046 21658702

[40] Yin M Yang D Lai S Yang H. Rheological properties of xanthan-modified fish gelatin and its potential to replace mammalian gelatin in low-fat stirred yogurt. LWT. (2021) 147:111643. 10.1016/j.lwt.2021.111643

[41] Kulmyrzaev AA Schubert H. Influence of KCl on the physicochemical properties of whey protein stabilized emulsions. Food Hydrocoll. (2004) 18:13–9. 10.1016/S0268-005X(03)00037-7

[42] Feng X Chen L Lei N Wang S Xu X Zhou G. Emulsifying properties of oxidatively stressed myofibrillar protein emulsion gels prepared with (–) -epigallocatechin-3-gallate and NaCl. J Agr Food Chem. (2017) 65:2816–26. 10.1021/acs.jafc.6b05517 28267324

[43] Tan Y Deng X Liu T Yang B Zhao M Zhao Q. Influence of NaCl on the oil/water interfacial and emulsifying properties of walnut protein-xanthan gum. Food Hydrocoll. (2017) 72:73–80. 10.1016/j.foodhyd.2017.05.031

[44] Fei Y Liu S Jian X Qiang L Fang W Sun D. Pickering emulsions stabilized solely by layered double hydroxides particles: the effect of salt on emulsion formation and stability. J Colloid Interf Sci. (2006) 302:159–69. 10.1016/j.jcis.2006.06.015 16842811

[45] Wu J Shi M Li W Zhao L Wang Z Yan X. Pickering emulsions stabilized by whey protein nanoparticles prepared by thermal cross-linking. Colloids Surf B. (2015) 127:96–104. 10.1016/j.colsurfb.2015.01.029 25660092

[46] Xu D Zheng B Che Y Liu G Cao Y. The stability, microstructure, and microrheological properties of *Monascus* pigment double emulsions stabilized by polyglycerol polyricinoleate and soybean protein isolate. Front Nutr. (2020) 7:543421. 10.3389/fnut.2020.543421 33385004 PMC7770174

[47] Dickinson E. Flocculation of protein-stabilized oil-in-water emulsions. Colloids Surf B. (2010) 81:130–40. 10.1016/j.colsurfb.2010.06.033 20667698

[48] Shen R Lin D Liu Z Zhai H Yang X. Particles for the stabilization of high internal phase Pickering emulsions by anti-solvent precipitation and their application in the delivery of curcumin. Front Nutr. (2021) 1:734620. 10.3389/fnut.2021.734620 34557512 PMC8454892

